# Decidual stromal cells drive CD16^+^ macrophages towards an immunoregulatory phenotype via extracellular matrix-adhesion molecule interaction during early pregnancy

**DOI:** 10.3389/fimmu.2025.1747323

**Published:** 2026-01-05

**Authors:** Hui-Li Yang, Jia-Wei Shi, Zhen-Zhen Lai, Xin Li, Chun-Jie Gu, Zi-Meng Zheng, Hong-Bo Zhao, Jiang-Feng Ye, Li Wang, Ting Peng, Ming-Qing Li

**Affiliations:** 1 Department of Reproductive Immunology, The International Peace Maternity and Child Health Hospital, School of Medicine, Shanghai Jiao Tong University, Shanghai, China; 2 Shanghai Key Laboratory of Embryo Original Diseases, The International Peace Maternity and Child Health Hospital, School of Medicine, Shanghai Jiao Tong University, Shanghai, China; 3 Department of Obstetrics and Gynecology, The First Affiliated Hospital of Ningbo University, Ningbo, China; 4 Department of Obstetrics and Gynecology, Fujian Medical University Union Hospital, Fuzhou, China; 5 Institute of Obstetrics and Gynecology, Hospital of Obstetrics and Gynecology, Fudan University, Shanghai, China; 6 Institute for Molecular and Cell Biology, Agency for Science, Technology and Research, Singapore, Singapore; 7 Center for Reproductive Medicine & Fertility Preservation Program, The International Peace Maternity and Child Health Hospital, School of Medicine, Shanghai Jiao Tong University, Shanghai, China; 8 Department of Obstetrics and Gynecology, Shanghai Changning Maternity & Infant Health Hospital, East China Normal University, Shanghai, China

**Keywords:** CD16, decidual stromal cell, extracellular matrix, macrophage, miscarriage

## Abstract

**Introduction:**

Dramatic alterations of the extracellular matrix (ECM), which can regulate cell behavior by binding to adhesion molecules and are intrinsically linked to immune regulation, occur in decidualization during early pregnancy. Decidual macrophages (dMφ) are a group of tissue-resident cells with an affinity for adhesion. An interactive dialogue occurs between decidual stromal cells (DSCs) and dMφ, however it remains unclear whether this process is associated with ECM-adhesion molecules. This study was conducted to investigate the cross-talk of DSC and CD16^+^ dMφ via extracellular matrix-adhesion molecule interaction in early pregnancy.

**Methods:**

Single-cell sequencing data from endometrial and decidual tissues were analyzed to elucidate the interactions between DSCs and dMφ. We assessed the levels of ECM components in the decidua at the tissue or cellular levels, and examined the expression of adhesion molecules and polarization molecules in CD16^+^ or CD16^-^ dMφ. Then we validated DSC-CD16^+^ Mφ interactions using the co-culture system. Finally, we evaluated the levels of ECM components in decidua and the expression of molecules in dMφ in patients with recurrent miscarriage (RM).

**Results:**

DSCs communicated with dMφ via ECM-adhesion molecules. Collagen IV, osteopontin (OPN) and hyaluronic acid (HA) derived from DSCs promoted the development of CD16^+^ dMφ and their differentiation toward an immunomodulatory phenotype via their receptors, which is beneficial for maintaining immune tolerance. In patients with RM, decidua exhibits weakened ECM-CD16^+^ dMφ regulatory link, which may be associated with the underlying pathogenesis.

**Discussion:**

This study confirms that DSC can regulate the immune status of CD16^+^ dMφ through the ECM-adhesion molecule axis, further elucidating the regulatory mechanisms of Mφ during decidualization. Exploration of therapeutic strategies based on ECM-receptor-mediated stroma-immune interactions holds promise as novel treatment approaches for miscarriage.

## 1 Introduction

The maternal-fetal interface consists of placental tissue and decidual tissue, within which components of the maternal immune system are present. The establishment of immune tolerance is essential for occurrence and establishment of a normal pregnancy. The interactive network among the decidua, trophoblast and immune cells plays an indispensable role in this process (1).

As an important component of the tissue microenvironment, extracellular matrix (ECM) serves as a vital bridge for intercellular communication. The ECM undergoes continuous degradation and reconstruction during decidualization, embryo implantation and placentation. The ECM performs both mechanical and biochemical functions, maintaining uterine structural integrity, facilitating embryo adhesion, and regulating trophoblast invasion into the endometrium during a healthy pregnancy (2). Stroma undergoes a profound transformation through decidualization, accompanied by alterations in cellular components including ECM. Abnormal ECM expression (e.g. collagens) and ECM remodeling disorders are associated with pathological pregnancy, such us recurrent miscarriage, gestational diabetes and preeclampsia (3).

Decidua serves as a primary site for immune cell aggregation and functions as a crucial location for immune interactions between the fetus and the mother (4). Macrophages account for approximately 20% of immune cells within the human decidua (5). Macrophages are cells with remarkable plasticity, exhibiting phenotypic switching and responding to environmental perturbations. During the different phases of gestation, macrophages undergo dynamic changes based on stimuli and mediators, differentiating to neither the classic M1 nor M2 subset (5). Accumulating evidence has indicated that M2 macrophages are the predominant phenotype in the decidua of early pregnancy (5, 8). Abnormalities in the proportion, phenotype, or function of decidual macrophages (dMφ) are closely associated with early pregnancy loss (6). Abnormalities in specific subpopulations of dMφ may lead to adverse pregnancy outcomes, such as recurrent miscarriage or preeclampsia (7, 8). The dMφ are co-regulated by the stroma, trophoblasts and other immune cells with mechanisms involving specific pathways such as metabolites, cytokines, growth factors and other immune signaling (9). The cross-talk between DSCs and dMφ has been the most extensively studied. Our recent study has found that Fructose-1,6-bisphosphate (FBP) from decidual stromal cells (DSCs) prevents pregnancy loss by inducing COX-2^+^ dMφ differentiation (10). However, the specific mechanisms underlying the interactive dialogue between DSC and dMφ in decidua where ECM undergoes significant changes still require further exploration.

ECM contains intrinsic biochemical and mechanical signals that respond to various physiological and pathological stimuli to regulate cellular phenotype and function (11). ECM can trigger intracellular signaling and regulate cellular behavior by binding to cell adhesion molecules such as integrins and glycoprotein (11). Once fully differentiated, DSCs begin secreting large quantities of ECM molecules, which induce significant changes in the composition and structure of the endometrial stroma (2). Decidual macrophages constitute a highly adhesive population of immune cells. High expression of adhesion molecules facilitates their local adhesion and residency. Our previous study has revealed that insufficient residency and reduced proportion of dMφ correlates with adverse pregnancy outcomes (6). DSCs and dMφ interactions may be related to the interactions between ECM and adhesion molecules. Previous studies have demonstrated that ECM components such as decorin and hyaluronan can regulate the polarization of dMφ (12, 13). However, the regulatory mechanisms by which ECM components derived from dramatically altered stromal cells during decidualization influence macrophages lack systematic investigation.

Here, we have analyzed the dialogue between DSCs and dMφ via ECM and adhesion molecules, further elucidating the regulatory mechanisms of macrophages during decidualization.

## 2 Materials and methods

### 2.1 Tissue collection

All tissues were collected from patients in The International Peace Maternity and Child Health Hospital of Shanghai Jiao Tong University from Feb 2025 to Aug 2025 with ethics committee approval (Approval No. GKLW-A-2025-020-01) and informed consent of the donors. Endometrial tissues of secretory phase (n=36) were collected from women of reproductive ages (29–38 years old) with normal pregnancy history, undergoing a hysteroscopic procedure with a pathological diagnosis of benign. These donors had not received any hormone-related treatment within the past six months. Decidual tissues were from women (n=63) with normal first-trimester pregnancy for selective termination (age, 21–35 years old; gestational age, 7–9 weeks) or patients (n=30) with unexplained recurrent miscarriage (age, 28–37 years old; gestational age, 6–9 weeks). The samples were collected in the operating room under strict aseptic conditions throughout the procedure and transported to the laboratory on ice within 30 minutes in Dulbecco’s modified Eagle’s medium (DMEM)/F-12 (HyClone, SH30023.01B) with 10% fetal bovine serum (FBS; Gibco, 26140-079) for further assays or cell isolation.

### 2.2 Cell isolation

Primary endometrial stomal cells (ESCs) and endometrial immune cells (EICs) from endometrial tissues were isolated and collected for flow cytometry analysis according to our previous protocols (14). DSCs and DICs were also obtained according to the experimental methods previously reported by us (6). ESCs or DSCs were cultured in DMEM/F-12 (HyClone, SH30023.01) containing 10% FBS (Gibco, 26140-079). DMφ from DICs were obtained through positive selection by an CD14^+^ cell isolation kit according to the reagent instructions (Miltenyi Biotec, 130–050-201).

### 2.3 scRNA-seq sequencing data analysis

Single-cell RNA sequencing (scRNA-seq) data were obtained from the NCBI Gene Expression Omnibus (GEO) database, GSE183837 (endometrium) and GSE194219 (decidua). Data sequencing and analysis was conducted by NovelBio Bio-Pharm Technology Co., Ltd. Singlecell dissociation (15). QuSAGE analysis was used to describe the relative pathway activation of specific genes in stromal cells and macrophages. Gene Ontology analysis (http://www.geneontology.org) was performed to analyze the enriched pathways of differentially expressed genes in DSC and ESC (false discovery rate [FDR] < 0.05). Cell communication analysis was based on CellPhoneDB (16). Based on the interaction and normalized cell matrices obtained through Seurat normalization, significant mean values and cell communication significance (p-value < 0.05) were calculated. Pathways of ligands from DSC and receptors from dMφ were enriched by KEGG pathway in gene set enrichment analysis.

### 2.4 Cell treatment and co-culture

Human monocyte cell line U937 from American Type Culture Collection (ATCC, CRL-3253) was cultured with RPMI-1640 medium (HyClone, SH30027.01) containing 10% FBS. U937 cells (5 × 10^5^/well in 6-well plate) were treated with recombinant human (rH) macrophage colony stimulating factor (CSF1, 50 ng/mL; Pepro Tech, AF-300-25-100) to induce M0 macrophages and both rH-CSF1 (50 ng/mL) and rH-IL10 (50 ng/mL; Pepro Tech, AF-200-10-100) for 6 days to induce decidual-like M2 macrophages according to previous report (17).

M2φ were treated with exogenous COL4A1 (10 ug/mL; Proteintech, Ag34402), OPN (1 ug/mL; ABclonal, RP00989), and HA (High Molecular Weight; 50μM, 100μM; R&D system, GLR002) for 48 hours. Gene silencing plasmid targeting *COL4A1* (si*COL4A1*)/*SPP1* (si*SPP1*) or control plasmid was from Shanghai Genechem Co., LTD. (China). DSCs were seeded in 6-well culture plates (5 × 10^5^ cells/well) and subsequently infected with si*COL4A1*/si*SPP1* or control plasmid. Twelve hours after infection, fresh complete culture medium was used to continue the culture. The infection efficiency was evaluated by RT-qPCR 96 hours after infection. DSCs were seeded in 6-well culture plates (5 × 10^5^ cells/well) and treated with 4-Methylumbelliferone (4-MU; 500 μM; MCE, HY-N0187), a hyaluronan synthase inhibitor or vehicle (1‰ DMSO) for 48 hours.

After 48 hours of culture of si*COL4A1*/si*SPP1*/Ctrl DSCs or 4-MU/vehicle-treated DSCs in 24‐well plate (1 × 10^5^ cells/well), M2φ (1 × 10^5^ cells/well) was then placed in the upper compartment of the transwell chamber inserts (0.4 μm aperture, 12 mm diameter; Corning) in the non‐contact transwell co‐culture unit. After 48‐hour co‐culture, macrophages were collected and flow cytometry assays were carried out to analyze the levels of CD16 and other polarization markers in them.

### 2.5 RT-qPCR

The transcription levels of *COL4A1* or *SPP1* in DSCs were verified by RT-qPCR according to the standard protocols (RR036A and RR820A; Takara). All values obtained were normalized to the values obtained for β-actin (*ACTB*). The primer sequences were synthesized by Sangon Biotechnology Co., Ltd. *COL4A1*: forward 5′ -GGGATGCTGTTGAAAGGTGAA-3′, reverse 5′ -GGTGGTCCGGTAAATCCTGG-3′; *SPP1*: forward 5′ -CTCCATTGACTCGAACGACTC-3′, reverse 5′ -CAGGTCTGCGAAACTTCTTAGAT-3′; *ACTB*: forward 5′ - GCCGACAGGATGCAGAAGGAGATCA-3′, reverse 5′ - AAGCATTTGCGGTGGACGATGGA-3′. The fold change of the gene expression was calculated using the change in cycle threshold value method (ΔΔCt).

### 2.6 Immunohistochemistry and Alcian blue staining

Paraffin sections were technically supported by Wuhan Servicebio Technology Co., Ltd. (China). Human endometrial or decidua samples were incubated with rabbit anti-human COL4A1 antibody (1:1000; Abcam, ab308360) or rabbit anti-human OPN antibody (1:1000; Abcam, ab214150) overnight at 4°C in a humid chamber. After washing three times with TBS (Beyotime, ST661), the sections were incubated and the signal was visualized with GTvision TMIII Immunohistochemical Detection Kit (Gene Tech, GK500710). The sections were counterstained with hematoxylin (Well Biotech, WH2013).

To detect the distribution of hyaluronic acid, de-waxed endometrial or decidua sample sections were incubated with Digestion Solution or Negative Solution at 37°C for 3 hours according to Hyaluronic Acid Stain Kit (Solarbio, G3710). After rinsed with running water for 5min, the sections were stained with Alican Stain Solution.

### 2.7 Enzyme linked immunosorbent assay

Primary ESCs and DSCs were cultured in 24-well plate with a density of 1 × 10^5^ cells for 24 hours and then the culture supernatant of ESCs or DSCs was harvested. The levels of COL4A1, OPN and HA of cell culture supernatant were detected by Human COL4A1 ELISA Kit (ELK Biotechnology, ELK2838), Human OPN ELISA Kit (ELK Biotechnology, ELK10182) and Human HA ELISA Kit (ELK Biotechnology, ELK10906) according to the standard directions.

### 2.8 Flow cytometry assay

EICs and dMφ were collected to detect the expression of CD45 (PE/CY5 anti-human CD45 antibody; BioLegend, 982324), CD14 (PE-CY7 anti-human CD14 antibody; BioLegend, 982510), CD16 (APC-CY7 anti-human CD16 antibody; BioLegend, 360710), polarization markers (PE anti-human CD86 antibody, BioLegend, 374206; PE anti-human CD209 antibody, BioLegend, 330108; APC anti-human HLA-DR antibody, BioLegend, 307610; FITC anti-IDO antibody, eBioscience, 11-9473-82) and adhesion markers (BV510 anti- CD49a antibody, BD Biosciences, 755208; FITC anti-human CD44 Antibody, BioLegend, 397518; FITC anti-LYVE1 antibody, eBioscience, 53-0443-82; APC anti-human Integrin αVβ3 antibody, BioLegend, 304416). DICs from normal pregnant women and women with RM were isolated to detect the expression of CD45 (BV 421 anti-human CD45 Antibody, 368522), CD14 (PE-CY7 anti-human CD14 antibody; BioLegend, 982510), CD16 (BV785 anti-human CD16 antibody; BioLegend, 360734), CD86 (FITC anti-human CD86 antibody; BioLegend, 374204), CD206 (APC anti-human CD206 antibody; BioLegend, 321110) and CD209 (PerCP/Cyanine5.5 anti-human CD209 antibody; BioLegend, 330110). CD16 (APC-CY7 anti-human CD16 antibody; BioLegend, 360710), CD86 (PE anti-human CD86 antibody, BioLegend, 374206), CD206 (APC anti-human CD206 antibody; BioLegend, 321110), CD209 (PerCP/Cyanine5.5 anti-human CD209 antibody; BioLegend, 330110) of M2φ treated with exogenous COL4A1, OPN or HA and collected from different co-culture system were also analyzed by flow cytometry assays. Flow cytometry was performed using a Beckman CytoFLEX S flow cytometer (Beckman) equipped with Becton CytExpert software. Data analysis was conducted using FlowJo V10 software.

### 2.9 Statistical analysis

The results were representatives of multiple experiments. All analyses were conducted by SPSS 25 (IBM). Normally distributed data with uniform variance were analyzed by a t test between two groups or one-way ANOVA test among multiple groups. Data were presented as mean ± standard error (SEM). P < 0.05 was considered to indicate a statistically significant difference.

## 3 Results

### 3.1 Decidualization is accompanied by active ECM organization

**Figure 1:**
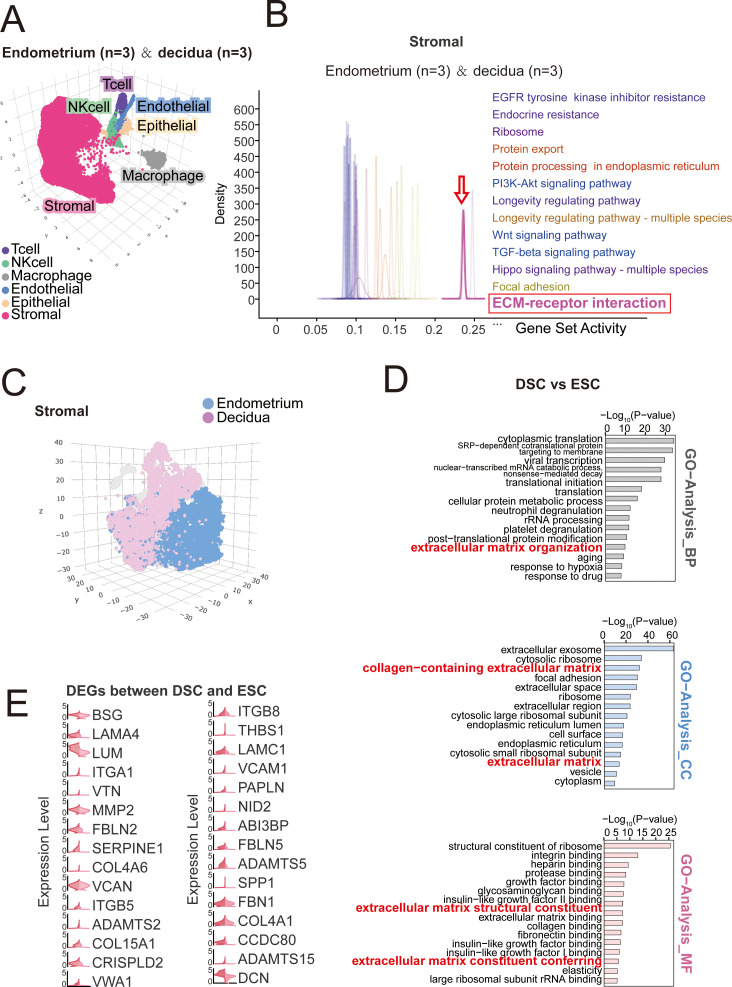
Decidualization is accompanied by active ECM organization. **(A)** Cell type identification of single cells from endometrium (n=3) and decidua (n=3) samples plotted in UMAP (3D) coordinates by scRNA-seq. **(B)** Enrichment geneset list in QUsage analysis of stromal cell population from **(A)**. **(C)** Presentation of stromal cells from endometrium (n=3) or decidua (n=3) in UMAP (3D) plot by scRNA-seq. **(D)** GO analysis of different genes in scRNA-seq database between DSCs and ESCs (CC: Cellular Component; BP: Biological Process; MF: Molecular Function). **(E)** Expression of ECM-related genes of DSCs and ESCs in Violin plots from scRNA-seq database.

**Figure 2:**
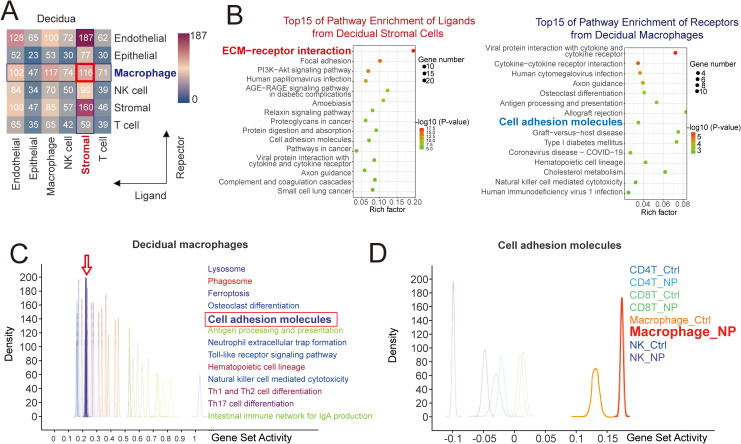
Cross-talk between stroma and macrophages via ECM-adhesion molecules is active. **(A)** Cell interaction analysis of different cell populations including endothelial cells, epithelial cells, macrophages, NK cells, stromal cells and T cells in decidua (n=3) from scRNA-seq database. **(B)** Pathways enrichment of ligands from decidual stroma cells (left) and receptors from decidual macrophages (right) in KEGG pathway analysis from scRNA-seq database. **(C)** Enrichment geneset list in QUsage analysis of decidual macrophage population from scRNA-seq database. **(D)** Expression of genes of adhesion molecules from different immune cells in endometrium (Ctrl, n=3) or decidua (NP, n=3) in QUsage analysis.

**Figure 3:**
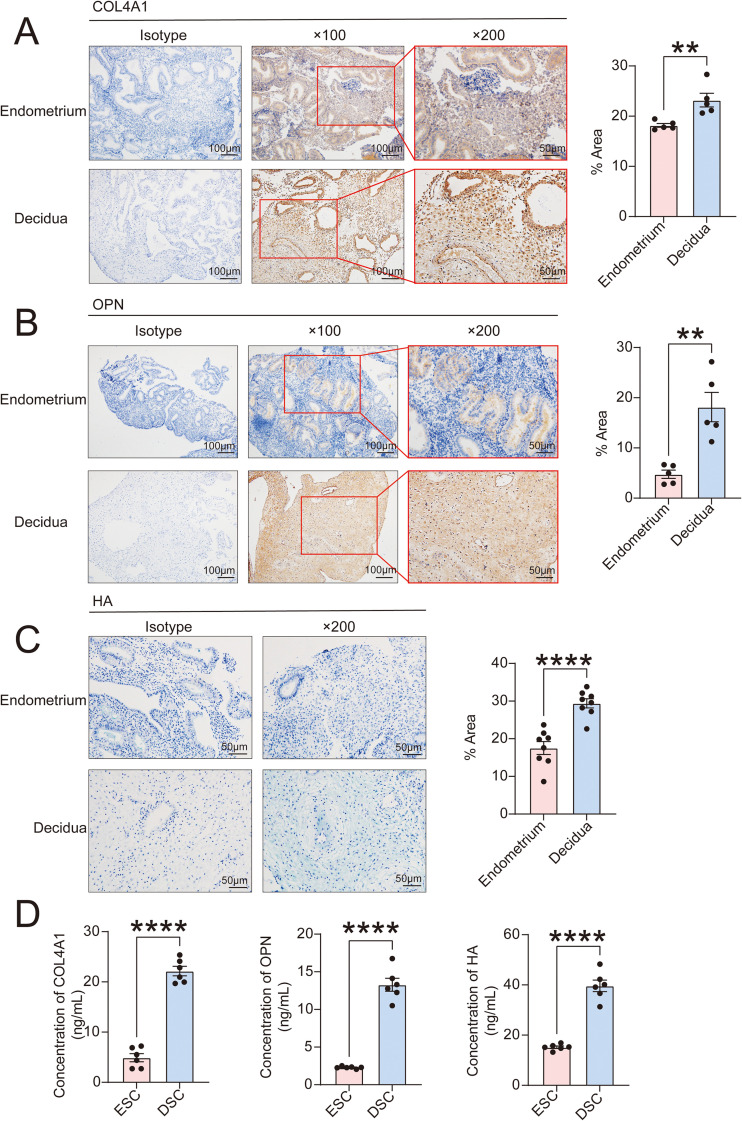
The decidua is rich in COL4A1, OPN and HA. **(A, B)** Compared the expression of COL4A1 **(A)** and OPN **(B)** in endometrium (n=5) and decidua (n=5) by Immunohistochemistry. Scale bar, 100 μm and 50 μm. **(C)** Compared the expression of HA in endometrium (n=8) and decidua (n=8) by Alcian blue staining assays. Scale bar, 100 μm. **(D)** The levels of COL4A1, OPN and HA from the culture supernatant of DSCs (n=6) or ESCs (n=6) were evaluated by ELISA. The data are presented as the mean ± SEM; two-tailed Student’s *t*-test; **P < 0.01, ****P < 0.0001.

Based on our single-cell sequencing data (15), stromal cells constituted the largest cellular component in both endometrial and decidual tissues (**Figure 1A**). Compared to other cell populations, genes associated with ECM-receptor interaction were highly enriched in stromal cells (**Figure 1B**). We performed data analysis on differentially genes in endometrial stromal cells (ESCs) and DSCs (**Figure 1C**). As showed in **Figure 1D**, GO enrichment analysis was performed using the R package to obtain information about biological process (BP), cellular component (CC), and molecular function (MF). Compared with ESCs, ECM-related pathways were significantly enriched in DSCs, such as ECM organization, collagen-containing ECM and ECM constituent conferring. In DSCs, genes associated with ECM organization and remodeling were generally significantly overexpressed (**Figure 1E**), suggesting that the matrix undergoes profound changes during decidualization. Since collagen, hyaluronic acid (HA), and osteopontin (OPN) have been reported in previous studies to be important ECM components regulating the immune state at the maternal-fetal interface (3, 13, 18), we focused on detecting and validating their expression in the decidua and endometrium. Combined with scRNA sequencing results, including alterations in ECM-related genes during decidualization and insights from cell communication signal analysis we observed subsequently (**Figure 2A**), we confirmed through Immunohistochemical or Alcian blue staining analysis that the levels of type IV collagen (COL4A1), osteopontin (OPN), and hyaluronic acid (HA) in normal early pregnancy decidua were significantly higher than those in secretory phase endometrium (**Figures 3A-C**). To further confirm that they can be synthesized from DSCs and transferred to the matrix to exert regulatory functions, we supplemented the study with ELISA assays (**Figure 3D**). Compared to ESCs, higher levels of COL4A1, OPN, and HA were detected in the culture supernatants of DSCs, which may play a crucial regulatory role in cell-to-cell communication within the matrix in decidualization.

### 3.2 DSCs and dMφ communicate via ECM-adhesion molecule interactions

In single-cell sequencing analysis of cell-cell communication pathways, over 100 interaction pathways were identified between as ligands on DSCs and receptors on dMφ (**Figure 2A**). Then, we performed enrichment analysis on genes associated with ligands and receptors (**Figure 2B**). Genes about ECM receptor interaction were significantly enriched in ligands from DSC, while cell adhesion molecule-related genes were significantly enriched in receptors from dMφ (**Figure 2B**). This result suggested that cross-talk between decidua and macrophages might occur via ECM-adhesion molecules. Furthermore, we identified a high expression of cell adhesion molecules in macrophages through gene set activity clustering analysis, consistent with their characteristics as tissue-resident macrophages (**Figure 2C**). Compared to endometrial macrophages (eMφ; Macrophge_Ctrl), dMφ (Macrophage_NP) exhibited higher expression levels of adhesion molecules, which may participate in interactions between DSCs (**Figure 2D**).

### 3.3 CD16^+^ Mφ exhibits a high proportion with immunoregulatory phenotype in decidua

**Figure 4:**
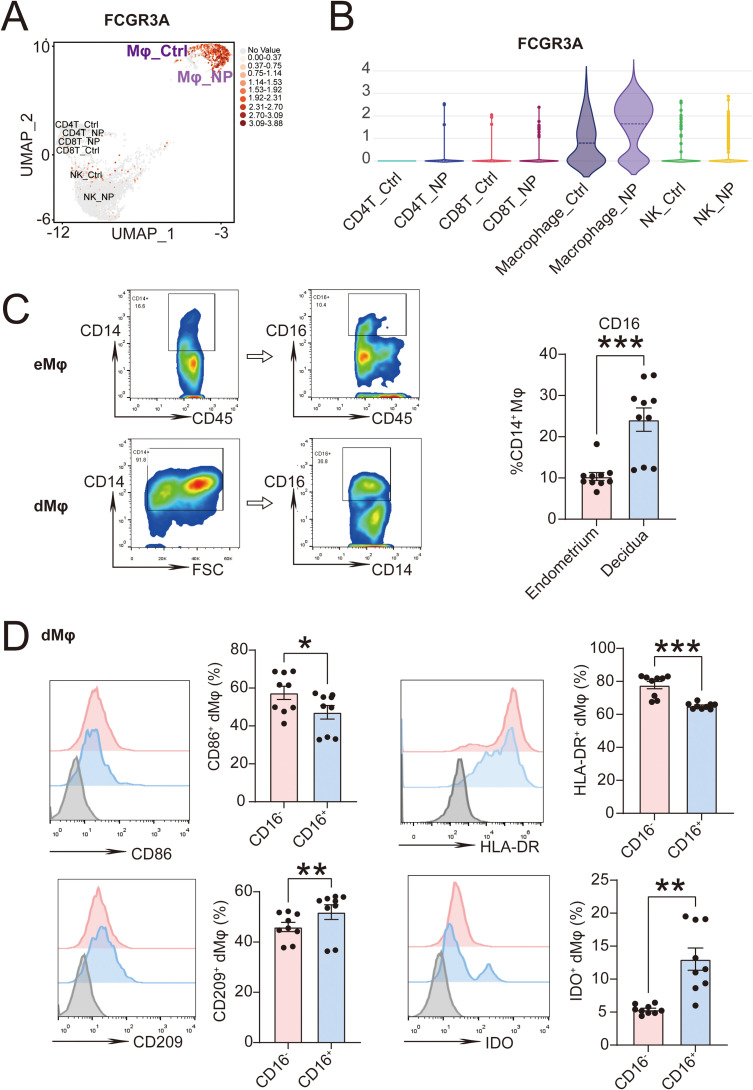
CD16^+^ macrophages constitute a high proportion in decidua with an immunoregulatory phenotype. **(A)** Expression of *FCGR3A* in immune cells from endometrium (Ctrl, n=3) or decidua (NP, n=3) in UMAP plots of scRNA-seq analysis. And the violin plots is shown in **(B)**. **(C)** Levels of CD16 in CD45^+^CD14^+^ macrophages from endometrium (n=10) or decidua (n=10) detected by flow cytometry assays. **(D)** Expression of CD86, HLA-DR, CD209 and CD206 in CD14^+^CD16^+^ or CD14^+^CD16^-^ dMφ (n=9) in flow cytometry assays. The data are presented as the mean ± SEM; two-tailed Student’s *t*-test; *P < 0.05, **P < 0.01, ***P < 0.001.

Through single-cell sequencing analysis (15), we have discovered that *FCGR3A* (gene encoding CD16) was primarily expressed in macrophages in both endometrium (Macrophge_Ctrl) and decidua (Macrophage_NP) in the UMAP visualization (**Figure 4A**). Compared with eMφ (Macrophge_Ctrl), there was an upward trend in *FCGR3A* expression in dMφ (Macrophage_NP) in the violin analysis diagram (**Figure 4B**). Confirmed by flow cytometry, CD16 expression on CD45^+^CD14^+^ dMφ was significantly higher than on CD45^+^CD14^+^ eMφ (**Figure 4C**). We then detected the polarized molecules in CD45^+^CD14^+^CD16^-^ dMφ and CD45^+^CD14^+^CD16^+^ dMφ by flow cytometry assays. CD16^+^ dMφ exhibited an immunoregulatory phenotype, characterized by higher expression of CD209 and IDO, along with lower levels of CD86 and HLA-DR compared to CD16^-^ dMφ (**Figure 4D**).

**Figure 5:**
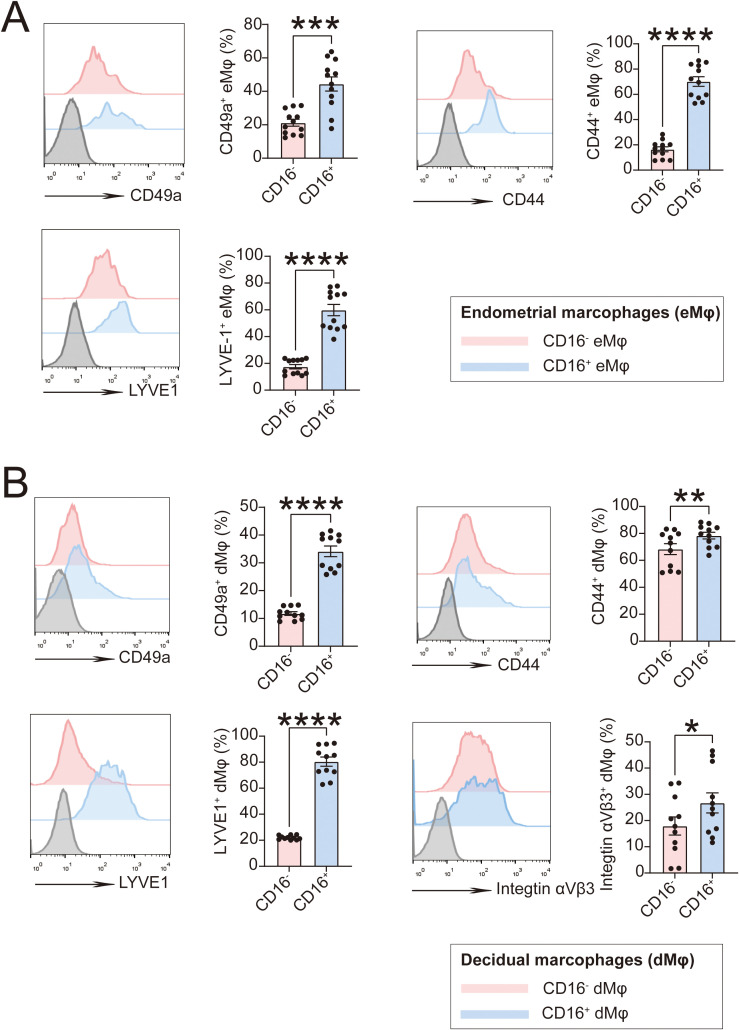
CD16^+^ macrophages from endometrium and decidua exhibit higher levels of adhesion molecules. **(A)** Expression of CD49a, CD44 and LYVE1 in CD45^+^CD14^+^CD16^+^ or CD45^+^CD14^+^CD16^-^ eMφ (n=12) in flow cytometry assays. **(B)** Expression of CD49a, CD44, LYVE1 and Integrin αVβ3 in CD14^+^CD16^+^ or CD14^+^CD16^-^ dMφ (n=11) in flow cytometry assays. The data are presented as the mean ± SEM; two-tailed Student’s *t*-test; *P < 0.05, **P < 0.01, ***P < 0.001, ****P < 0.0001, ns.

Specifically, we found in flow cytometry that CD45^+^ CD14^+^CD16^+^ Mφ in both endometrium (**Figure 5A**) and decidua (**Figure 5B**) exhibited higher expression of adhesion molecules compared with CD45^+^ CD14^+^CD16^-^ Mφ, suggesting a tendency toward tissue residency. Here, we focused on detecting CD49a (collagen IV receptor), CD44 and LVYE1 (HA receptor), and integrin αVβ3 (OPN receptor), whose expression levels in CD16^+^ dMφ were significantly higher than in CD16^-^ dMφ (**Figure 5B**). This suggests that ECM derived from the decidua may exert regulatory effects of CD16^+^ Mφ via these adhesion molecules.

### 3.4 COL4A1, OPN and HA derived from DSC promote the development of CD16^+^ dMφ

**Figure 6:**
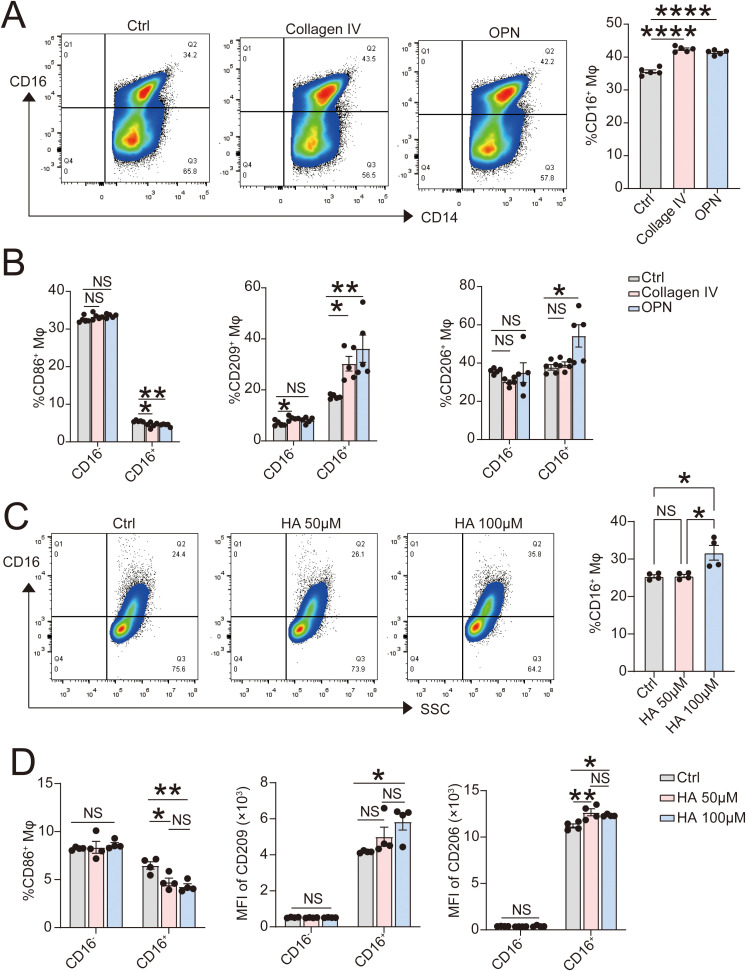
Exogenous COL4A1, OPN and HA promote development of immunomodulatory CD16^+^ Mφ. **(A, B)** After 48 hours of stimulation with exogenous COL4A1 (10 ug/mL), OPN (1 ug/mL) or vehicles, the expression levels of CD16 on U937-induced Mφ (**A**; n=5) and the polarization-related molecules CD86, CD209 and CD206 on CD16^-^ or CD16^+^ Mφ (**B**; n=5) were detected by flow cytometry. **(C, D)** After 48 hours of stimulation with exogenous HA (0, 50, 100 μM), the expression levels of CD16 on U937-induced Mφ (**C**; n=4) and CD86, CD209 and CD206 on CD16^-^ or CD16^+^ Mφ (**D**; n=4) were detected by flow cytometry. The data are presented as the mean ± SEM; oneway ANOVA test or two-tailed Student’s t-test; *P < 0.05, **P < 0.01, ****P< 0.0001, NS, no significant difference.

U937 cells were induced into dMφ-like macrophages using the previously mentioned method (17). Exogenous COL4A1 (10 ug/mL) and OPN (1 ug/mL) for 48 hours promoted CD16 expression on dMφ-like macrophages (**Figure 6A**) in flow cytometry assays. As shown in **Figure 5B**, the higher levels of receptors of COL4A1 and OPN were detected on CD16^+^ Mφ. The regulatory effects of COL4A1 and OPN on the polarized molecules in CD16^+^ Mφ were more pronounced in **Figure 6B**. Specifically, COL4A1 (10 ug/mL) and OPN (1 ug/mL) for 48 hours significantly downregulated CD86 expression on CD16^+^ Mφ while upregulating CD206 or CD209 expression on them. However, this effect was not significant on CD16^-^ Mφ. We then treated dMφ-like macrophages with high molecular weight HA at different concentrations (0, 50, 100μM) for 48 hours. In the high-concentration HA treatment group (100μM), the proportion of CD16^+^ macrophages highly expressing CD206 and CD209 significantly increased by flow cytometry (**Figures 6C, D**).

**Figure 7:**
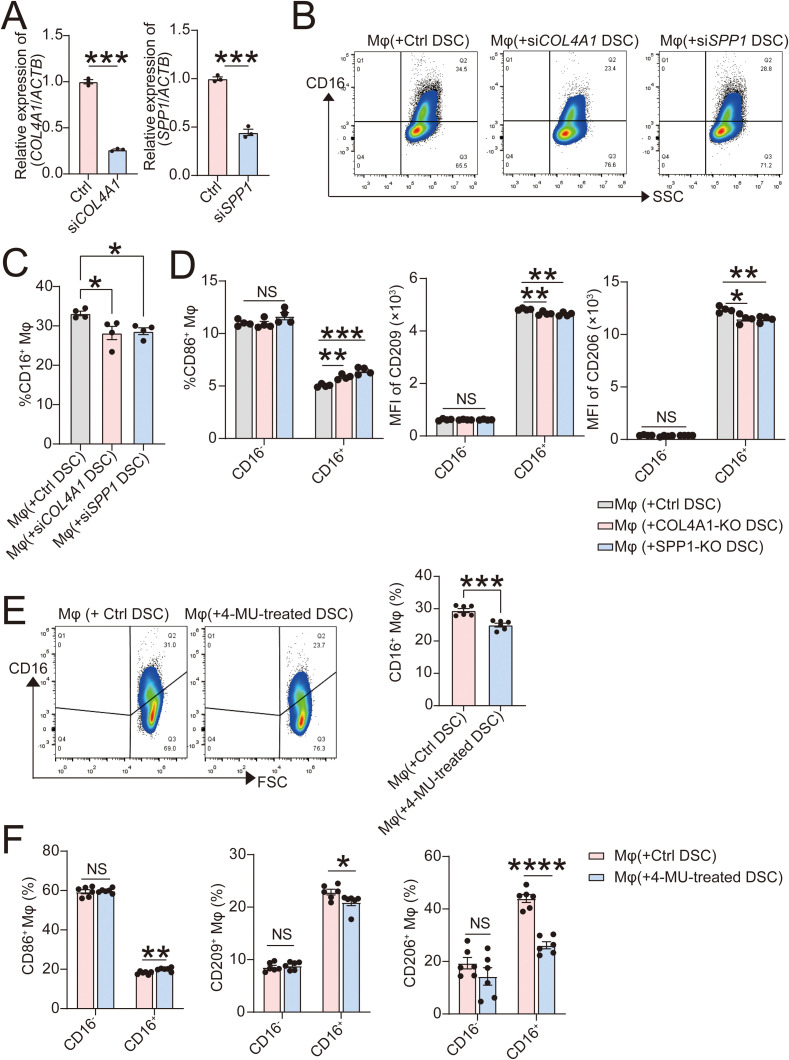
CD16^+^ Mφ in the co-culture system are suppressed with the inhibition of COL4A1, OPN and HA in DSCs. **(A)** DSCs were transfected by plasmid of siRNA targeting COL4A1 (si*COL4A1*; n=3), SPP1 (si*SPP1*; n=3) or control plasmids (n=3) for 72 hours and the efficacy was verified by RT-qPCR. **(B-D)** After co-cultured with *COL4A1*-silenced DSCs, *SPP1*-silenced DSCs or control DSCs for 48 hours, CD16 expression of U937-induced Mφ (**B, C**; n=4) and the polarization markers (CD86, CD209 and CD206) of CD16^-^ or CD16^+^ macrophages (**D**; n=4) were explored by flow cytometry. **(E, F)** After co-cultured with 4-MU (a hyaluronic Acid synthesis inhibitor, 500 μM) or vehicle treated DSCs for 48 hours, CD16 expression of U937-induced Mφ (**E**; n=6) and the polarization markers of CD16^-^ or CD16^+^ macrophages (**F**; n=6) were explored by flow cytometry. The data are presented as the mean ± SEM; oneway ANOVA test or two-tailed Student’s *t*-test; *P < 0.05, **P < 0.01, ***P < 0.001, ****P < 0.0001, ns, NS significant difference.

Subsequently, we further validated this result by a co-culture system. Following co-culture with si*COL4A1* or si*SPP1* DSCs (gene knockout efficiency was validated by RT-qPCR in **Figure 7A**) for 48 hours, the proportion of CD16^+^ macrophages from the co-culture system significantly decreased (**Figures 7B, C**), accompanied by upregulation of CD86 and downregulation of CD206 or CD209 (**Figure 7D**) assessed by flow cytometry. Similarly, when HA synthesis was inhibited by 4-MU (500 μM for 48 hours) in DSCs, CD16 expression on macrophages in the co-culture system was suppressed (**Figure 7E**), leading to pro-inflammatory differentiation characterized by higher expression of CD86 and lower expression of CD206 and CD209 (**Figure 7F**).

### 3.5 Patients with recurrent miscarriage exhibit weakened interactions between DSCs and CD16^+^ dMφ

Dysfunction of the decidualized DSCs and abnormal expression of related genes correlate with adverse pregnancy outcomes, particularly early pregnancy failure (19). Specifically, abnormal interactions between DSCs and dMφ also hinder the establishment and maintenance of normal pregnancy, leading to recurrent miscarriage (RM) and other complications (10). We then assessed the ECM synthesis of decidua and CD16 expression of dMφ from RM patients to determine whether these alterations are associated with the disease.

**Figure 8:**
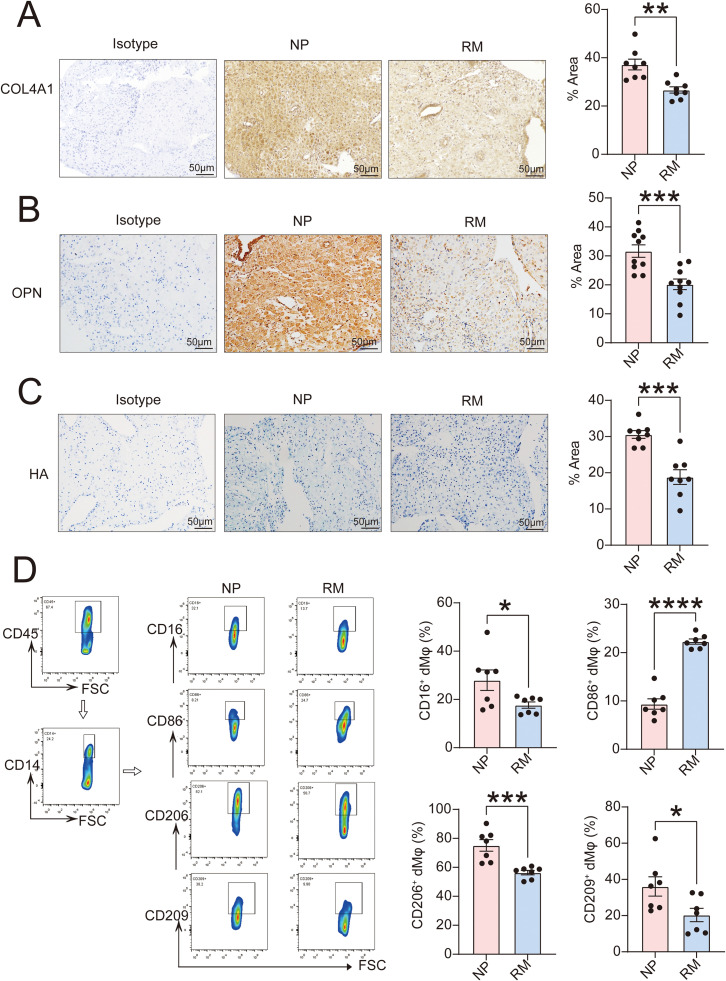
Patients RM exhibit weakened interactions between DSCs and CD16^+^ dMφ. **(A)** Compared the expression of COL4A1 in decidua from women with normal pregnancy (NP, n=8) or recurrent miscarriage (RM, n=8) by Immunohistochemistry. Scale bar, 50 μm. **(B)** Compared the expression of OPN in decidua from women with NP (n=10) or RM (n=10) by Immunohistochemistry. Scale bar, 50 μm. **(C)** Compared the expression of HA in decidua from women with NP (n=8) or RM (n=8) by Alcian blue staining assays. Scale bar, 50 μm. **(D)** Levels of CD16, CD86, CD206 and CD209 in CD45^+^CD14^+^ dMφ from women with NP (n=7) or RM (n=7) by flow cytometry assays. The data are presented as the mean ± SEM; two-tailed Student’s *t*-test; *P < 0.05, **P < 0.01, ***P < 0.001, ****P < 0.0001.

In Immunohistochemical or Alcian blue staining experiments, we detected significantly reduced levels of target ECM components in the decidua of RM patients compared to the normal pregnancy group (NP), including COL4A1 (**Figure 8A**), OPN (**Figure 8B**), and HA (**Figure 8C**). Through flow cytometry, we found that RM patients exhibited reduced CD16 expression in CD45^+^CD14^+^ dMφ, representing an immune-activated phenotype characterized by higher levels of CD86 and lower levels of CD206 and CD209 (**Figure 8D**). The above findings suggest that insufficient interaction between ECM-deficient DSCs and CD16^+^ dMφ may be associated with the occurrence of RM, which requires further exploration.

## 4 Discussion

For a long time, ECM was overlooked as an inert scaffold. It is now recognized as a highly dynamic partner of the immune system. Its rich protein components and immunologically active molecules also play an indispensable role in immune regulation under both homeostatic and pathological conditions. Conversely, the immune system maintains the homeostasis of the microenvironment within the matrix and restores its integrity following injury (20). From a reproductive immunology perspective, previous studies have demonstrated that ECM at the maternal-fetal interface may support pregnancy by exerting immunoregulatory effects (3). Here, our study further confirmed the regulatory role of the ECM in decidual immunity. We proposed multiple ECM components as bridges facilitating communication between DSCs and dMφ. COL4A1, OPN and HA derived from DSCs positively regulate the development of CD16^+^ Mφ in decidua and maintain their regulatory phenotype.

Most reports have demonstrated that dMφ with specific phenotypes play a pivotal role in regulating the decidual microenvironment (8). Here, we have confirmed the presence of CD16^+^ Mφ in decidua through single-cell sequencing analysis and flow cytometry, consistent with previous report (21). The phenotype of tissue-resident macrophages is subject to multifaceted regulation by their surrounding environment. ESCs significantly promoted CD16^+^CD14^hi^ macrophages with an immunoregulatory phenotype in peritoneal during ovarian endometriosis (22). In pregnancy, trophoblast induces monocytes differentiation into CD14^high^/CD16^high^ macrophages expressing immunoregulatory genes representative of an M2-like macrophage (21). In the current study, DSCs emerge as the key regulator of CD16^+^ dMφ, promoting the differentiation and development of M2-polarized CD16^+^ Mφ through ECM-adhesion molecule interactions. CD16^+^ Mφ highly express adhesion molecules in both endometrium and decidua, while the proportion of CD16^+^ dMφ is significantly higher than that of CD16^+^ eMφ. This suggests that CD16^+^ dMφ may originate partly from the endometrial resident population and partly from peripheral monocytes, with the latter being differentiated by the action of DSCs and trophoblasts before becoming locally retained in decidua.

Collagen at the maternal fetal interface produced by trophoblast and DSC promotes immune tolerance of DICs (3). Collagen IV, one of the major components of basal membranes, is selectively upregulated during the menstrual cycle and decidualization, when its deposition increases around the spiral arteries, highlighting its role in decidual vascular remodeling (23, 24). Lv et al. observed that collagen VI within the nerve conduit could induce polarized macrophage toward the M2 phenotype, and then promote nerve regeneration and functional recovery (25). Our study further reveals the regulatory role of collagen IV on dMφ. We found that COL4A1 derived from DSCs can promote the development of CD16^+^ immunoregulatory dMφ by binding to receptors on dMφ, such as CD49a.

OPN (also known as secreted phosphoprotein 1/SPP1), which can be found in ECM and binds integrins to mediate cell-cell and cell-ECM communication to promote cell adhesion, migration, and differentiation. OPN expression is upregulated in the endometrium and has received considerable interest as a key player during implantation and placentation (26, 27). Progesterone induces and the conceptus further stimulates OPN in uterine glands and stroma (28). OPN promotes M2 polarization of macrophages through the JAK2/STAT3 signaling pathway (29). Similarly, we found that at the maternal-fetal interface during early pregnancy, OPN derived from DSCs can also promote the differentiation of CD16^+^ dMφ toward an immunoregulatory phenotype via its receptor integrin αVβ3. The underlying mechanism may be associated with the JAK2/STAT3 signaling pathway, which warrants further investigation.

Research findings on the role of HA in normal pregnancy are frequently reported. HA is involved in the natural selection of human embryos at implantation (30). HA at maternal fetal interface promotes the invasion and proliferation of Tros as well as the growth of decidual stromal cells (31, 32). It was demonstrated that low-fitness embryos secrete high molecular weight hyaluronic acid (HMWHA), which, upon binding to CD44 on uNK cells, blocks the targeting and elimination of stressed/senescent cells (30). Higher secretion of HA by Tros could induce M2 polarization of dMφ by interacting with CD44 (13). When treating macrophages with different concentrations of hyaluronic acid, results showed that high concentrations of HA promote the M2-like phenotype of CD16^+^ macrophages. The primary mechanism involves the interaction of HA from DCS and high-level receptors on CD16^+^ dMφ, including CD44 and LVYE1. The specific molecular mechanisms require further investigation. These results enrich greatly the theoretical understanding of the regulatory mechanisms of maternal-fetal interface HA on dMφ.

The ECM and immune cells are mutually dependent, and exploring their intricate relationship holds promise for treating diseases and promoting healthy aging (20). A better understanding of the immune-regulatory mechanisms driven by ECM molecules would also be crucial considering that stromal remodeling represents a world to be explored for the identification of novel molecular markers for infertility and pregnancy diseases (2). ECM remodeling may be investigated as a therapeutic target for infertility and pregnancy disorders. A human ECM containing angiogenic and immunoregulatory cytokines is shown to induce *in vitro* and *in vivo* angiogenesis within engineered biomaterials while inhibiting implant fibrosis (33). The injection of HA during the embryo transfer is nowadays explored as a strategy to improve embryo implantation in IVF cycles (34). Similarly, further development and exploration of therapeutic strategies based on ECM-receptor-mediated stroma-immune interactions for regulating pregnancy-related disorders, such as unexplained recurrent miscarriage or implantation failure, holds promise as novel treatment approaches.

## Data Availability

The datasets presented in this study can be found in online repositories. The names of the repository/repositories and accession number(s) can be found in the article/supplementary material.
